# Parents' Awareness and Knowledge of Testicular Torsion in the Western Region of Saudi Arabia

**DOI:** 10.7759/cureus.36884

**Published:** 2023-03-29

**Authors:** Nedaa Alsulaimani, Ethar A Alsulami, Raghad E Saleh, Rawan S Alsamli, Fatoon M Almowallad, Renad T Alhazmi, Mohammed Ageel

**Affiliations:** 1 Department of Medicine, Faculty of Medicine, Umm Al-Qura University, Makkah, SAU; 2 Department of Surgery, Faculty of Medicine, Umm Al-Qura University, Makkah, SAU

**Keywords:** saudi arabia, parental awareness, children, ischemia, testis, testicular torsion

## Abstract

Introduction

Testicular torsion (TT) is the most common urological emergency in children that requires immediate intervention. The prognosis of testicular torsion depends on the patient's time of presentation to the emergency department (ED), as well as on the time at which the diagnosis is established and treatment is initiated. Raising public awareness of testicular torsion, particularly among parents, plays a crucial role in preventing delay in presentation and reducing the frequency of orchiectomy. To this end, the study is designed to assess the level of awareness and knowledge of testicular torsion, as well as the response of parents to the scrotal pain of their children.

Methods

A descriptive cross-sectional study was conducted among parents in the western region of Saudi Arabia. Data was collected between the 23rd of November and the 22nd of December 2022. A simple random sampling technique was implemented. The data was collected and analyzed using SPSS software (IBM Corp., Armonk, NY).

Results

A total of 394 parents participated in this study. It showed that 13.5% of parents reported having a child with a previous experience of pain in the scrotum. Of them, only 25.4% previously heard about torsion of the testicles. Only 68.8% and 76.6% correctly reported that if the child complains of pain in the scrotum during working hours or over the weekend, they will drive him to the hospital immediately. Good knowledge about TT was significantly higher among children's fathers compared to mothers (66.7% vs. 33.3%) (p≤0.05).

Conclusion

Testicular torsion (TT) represents significant morbidity among male patients and early identification is crucial to avoid the need for orchiectomy and all its prominent physical and psychological consequences. To improve children's well-being, we need to raise parents' awareness of TT and the potential future implications of this critical condition as it is not widely known in our community. Further studies evaluating the knowledge regarding testicular torsion among preadolescent and adolescent boys are warranted.

## Introduction

Acute scrotum pain can have several different etiologies, such as ischemia, trauma, infections, inflammation, referred pain, or idiopathy. However, the prevalence and incidence of these causes differ significantly by age group, showing that twisting of the testicular appendages or spermatic cord is significantly more prone to be the reason for acute scrotal pain in children [1].

Testicular torsion (TT) is the most common pediatric urological emergency that necessitates immediate intervention [2]. It is characterized by a longitudinal twisting of the spermatic cord, which restricts blood supply to the testis and results in ischemia of scrotal tissue which may alter testicular function temporarily or permanently, affects hormone production, cause eventual infertility, and in late presentation, may lead to orchiectomy [3,4].

Intravaginal torsion is the cause of the majority of TT cases in children. Bell clapper deformity is the term used to describe the anatomical condition that allows for intravaginal torsion. It is a congenital malformation in which the tunica vaginalis envelops the testis rather than attaching to the epididymis and the posterior surface of the testis, allowing the testis to rotate along a longitudinal axis [5]. It results in a 42% orchiectomy rate in males, accounting for up to 15% of pediatric acute scrotal pain. In the past, testicular torsion has been estimated to affect 3.8 out of 100,000 boys under the age of 18 [6].

All males who report scrotal or lower abdomen pain, scrotal enlargement, nausea, or vomiting should be evaluated for TT [4]. A high-ridden testicle with an absent cremasteric reflex may be discovered on physical examination [7]. Detorsion within four to eight hours after symptom onset has typically been considered to be the ideal window of opportunity to salvage testicular function in individuals with testicular torsion [2,8]. The prognosis of testicular torsion depends on the patient's presentation time to the emergency department (ED) and the time at which the diagnosis is established and treatment is initiated [6].

Orchidectomy significantly affects boys, and this influence cannot be neglected [9]. Raising public awareness of testicular torsion, particularly among parents, plays a crucial role in preventing delay in presentation and reducing the frequency of orchiectomy [10]. That is why it is necessary to evaluate what parents believe and how they will respond to it. A previous prospective study showed that an estimated 96% of parents whose kids experienced acute scrotal pain think it's critical to raise awareness of this condition [9].

Studies have been published on parents' awareness of testicular torsion in Saudi Arabia and around the world [8-12]. However, no studies have been conducted in the western region of Saudi Arabia to address parental knowledge and awareness of TT. To this end, the study is designed to assess the level of awareness and knowledge of testicular torsion, as well as the response of parents to the scrotal pain of their children among parents in the western region of Saudi Arabia; hopefully, this will work to reduce prehospital delays and increase the likelihood that testicles can be saved.

## Materials and methods

This is a descriptive cross-sectional study conducted among parents in the west of Saudi Arabia. Data was collected between the 23rd of November and the 22nd of December 2022. In total, 435 individuals consented to take part in this study. The inclusion criteria were all parents of all nationalities in the four administrative regions: Makkah, Jeddah, Madinah, and Taif. We excluded the parents who are working in the medical field (physicians, nurses, technicians), participants residing outside the western region, and parents of boys with a history of testicular torsion, genitourinary malformations, or history of cryptorchidism repair. After the exclusion criteria were applied, 394 parents were included.

An online structured, modified questionnaire was developed based on the reviewed literature [8]. The survey included three parts: the first asked about sociodemographic details, the second inquired about how parents responded to their children’s acute scrotal pain, and the third asked about parents' knowledge of TT and where they learned about it.

Ten questions were used to test knowledge; for seven of the questions, there was only one right response, earning a score of "1". A score of "0" was assigned for the incorrect response. For symptoms, risk factors, and complications of testicular torsion, every right answer was given a score of "1" leaving a total knowledge score ranging from 0-18. A participant's knowledge level was classified as "low" if they got fewer than 50% of the questions right, "fair" if they got between 50% and 75% of the questions right, and "good" if they got more than 75% of the questions right.

The completed questionnaires were analyzed by using (SPSS) version 26 (IBM Corp., Armonk, NY). Qualitative data were expressed as numbers and percentages to assess the relationship between variables, and the Chi-square test (χ2) was used. Quantitative data were expressed as mean and standard deviation (mean ± SD). Spearman's test was used for correlation analysis, and a p-value of less than 0.05 was regarded as statistically significant.

## Results

A total of 394 parents participated in this study; In 17.5%, ages ranged from 45-49 years, 91.1% were of Saudi nationality, and 40.6% were from Jeddah city. The majority (70.8%) were the children's mothers. Most of the participants (72.8%) received a university-level education, 60.2% were employed, 55.1% had one to two daughters, and 60.7 % had one to two sons (Table 1).

**Table 1:** Distribution of studied parents according to their demographic data and number of daughters and sons (n=394)

Variables		Number (n) (%)
Age	18-24	39 (9.9)
	25-29	55 (14)
	30-34	63 (16)
	35-39	50 (12.7)
	40-44	62 (15.7)
	45-49	69 (17.5)
	≥50	56 (14.2)
Nationality	Saudi	359 (91.1)
	Non-Saudi	35 (8.9)
City	Taif	49 (12.4)
	Madinah	85 (21.6)
	Jeddah	160 (40.6)
	Makkah	100 (25.4)
Relation to the child	Father	115 (29.2)
	Mother	279 (70.8)
Education level	Illiterate	2 (0.5)
	Primary	2 (0.5)
	Middle	17 (4.3)
	Secondary	86 (21.8)
	University	287 (72.8)
Employment	No	157 (39.8)
	Yes	237 (60.2)
The number of daughters	1-2	217 (55.1)
	3-4	98 (24.9)
	≥5	17 (4.3)
The number of sons	1-2	239 (60.7)
	3-4	99 (25.1)
	≥5	13 (3.3)

**Figure 1:**
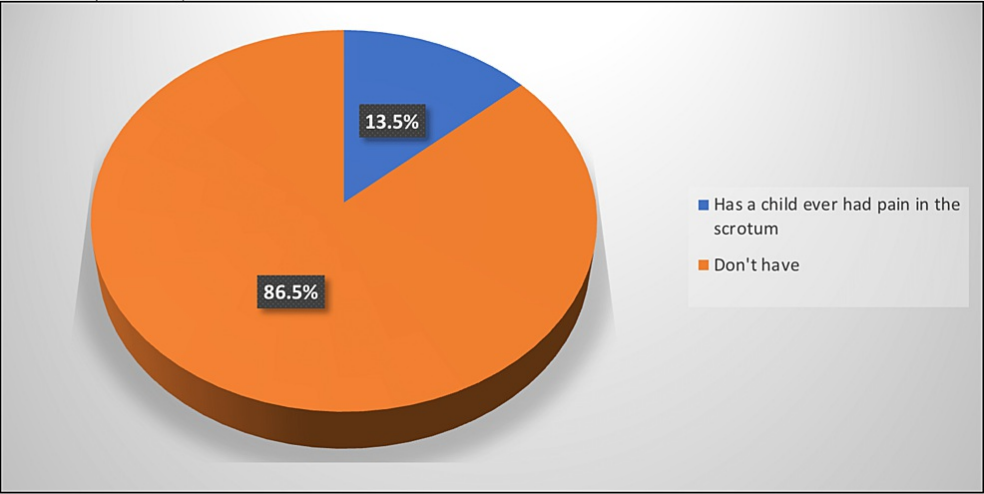
Percentage distribution of parents according to having a child experienced pain in the scrotum (n=394)

**Figure 2:**
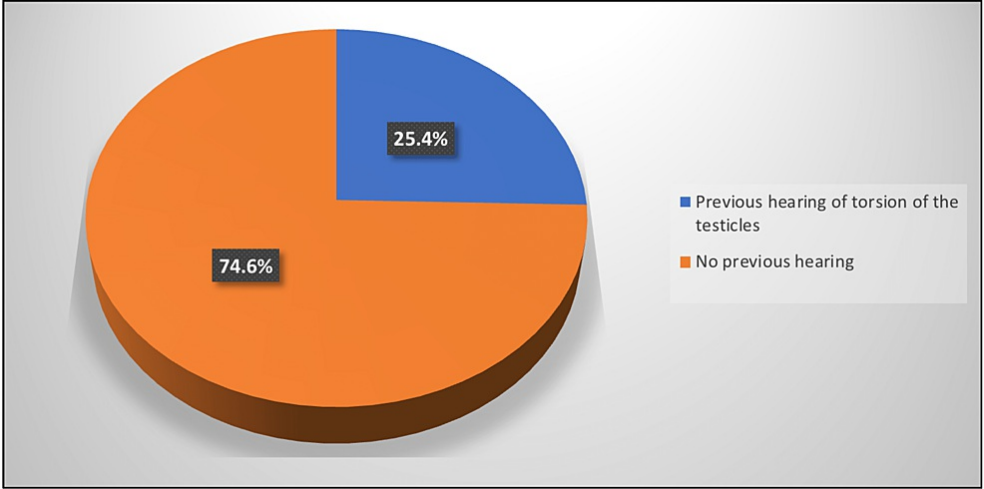
Percentage distribution of parents according to previous hearing about torsion of the testicles (n=394)

Our analysis showed that only 13.5% of parents reported having a child with a previous experience of pain in the scrotum (Figure 1), Of them, only 25.4% previously heard about torsion of the testicles (Figure 2).

The parents' knowledge about items related to torsion of the testicles is illustrated in Table 2. It showed that 68.8% and 76.6% correctly reported that if the child complains of pain in the scrotum during working hours or over the weekend, they will drive him to the hospital immediately. Only 6.3% reported that what makes them think this pain is a serious condition is their previous knowledge about how serious scrotal pain is. 

**Table 2:** Parents' responses to knowledge items related to torsion of the testicles (n=394) * correct answer

Variable		No. (%)
If your child complains of pain in the scrotum (during working hours), what will you do?	Will arrange an appointment with the urology clinic	44 (11.2)
	Put ice on it and try some home remedies	41 (10.4)
	Give him over-the-counter medication	38 (9.6)
	Drive him to the hospital immediately*	271 (68.8)
If your child complains of pain in the scrotum (over the weekend), what will you do?	Will arrange an appointment with the urology clinic	54 (13.7)
	Put ice on it and try some home remedies	17 (4.3)
	Give him over-the-counter medication	21 (5.3)
	Drive him to the hospital immediately*	302 (76.6)
At the time, what makes you think this pain is a serious condition?	If you try at home to put ice on or give him medication and the pain was not relieved by it	31 (7.9)
	If the pain did not relieve in a short time	189 (48)
	Severe pain and the appearance of the testis	132 (33.5)
	Your previous knowledge about how serious scrotal pain is*	25 (6.3)
	I don't think this pain seems serious	17 (4.3)
Do you know when the symptoms of testicular torsion appear (extremely severe pain)?	Immediately*	84 (21.3)
	Hours	41 010.4)
	Few days	20 (5.1)
	I don’t know	249 (63.2)
Torsion of the testicles is more common between ages:	Children (0-12)	104 (26.4)
	Adolescents (12-18)*	10 (2.5)
	Adults (older than 18)	56 (14.2)
	I don’t know	224 (56.9)
Symptoms of testicular torsion	Scrotal pain*	172 (43.7)
	Fever	58 (14.7)
	Swelling of the scrotum*	146 (37.1)
	Nausea	13 (3.3)
	Headache	9 (2.3)
	Abdominal pain*	48 (12.2)
	Vomiting	13 (3.3)
	Change in the color of the skin surrounding the scrotum*	75 (19)
	I don’t know	154 (39.1)
Do you know the risk factors for testicular torsion?	Age*	20 (5.1)
	Anatomic malformation*	58 (14.7)
	Previous testicular torsion*	42 (10.7)
	Family history of testicular torsion*	29 (7.4)
	Related to activity, trauma, during sleep	50 (12.7)
	I don’t know	235 (59.6)
Do you know the complications of testicular torsion?	Death of the testicle*	54 (13.7)
	Lower fertility*	95 (24.1)
	Infertility*	66 (16.8)
	No complications	5 (1.3)
	I don’t know	217 (55.1)
Do you know what is the appropriate time for surgical intervention?	Within four to six hours*	70 (17.8)
	With 12 hours	35 (8.9)
	I don’t know	289 (73.4)

About 21% (21.3%) knew that symptoms of testicular torsion appear immediately, and only 2.5% knew that torsion of the testicles is more common between 12-18 years. The correct known symptoms of TT by parents were scrotal pain (43.7%), swelling of the scrotum (37.1%), change in the color of the skin surrounding the scrotum (19%), and abdominal pain (12.2%). The correct known risk factors of TT by parents were anatomic malformation (14.7%), previous testicular torsion (10.7%), family history of testicular torsion (7.4%), and age (5.1%).

The correct known complications of testicular torsion by parents were lower fertility (24.1%), infertility (16.8%), and death of the testicle (13.7%). Only 17.8% of the participants knew that the appropriate time for surgical intervention is within four to six hours.

**Figure 3:**
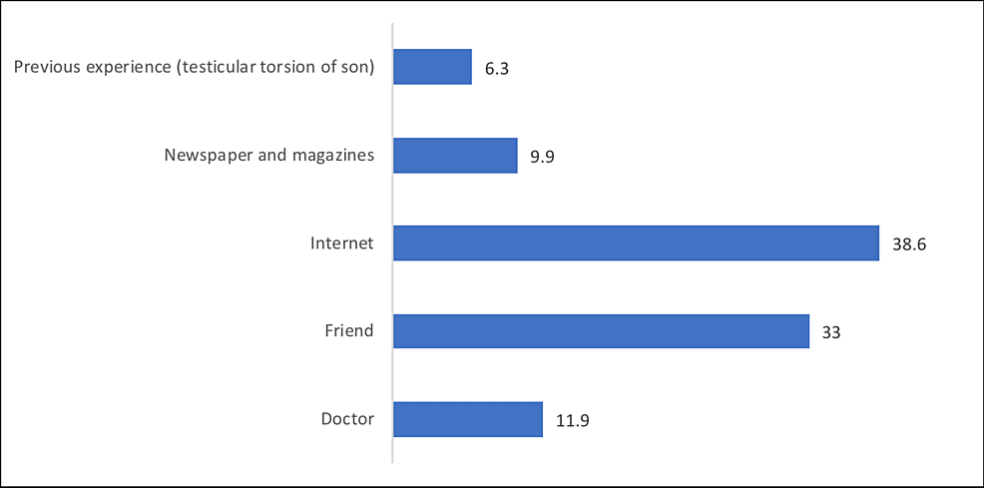
Percentage distribution of parents according to sources of information about testicular torsion (n=394)

**Figure 4:**
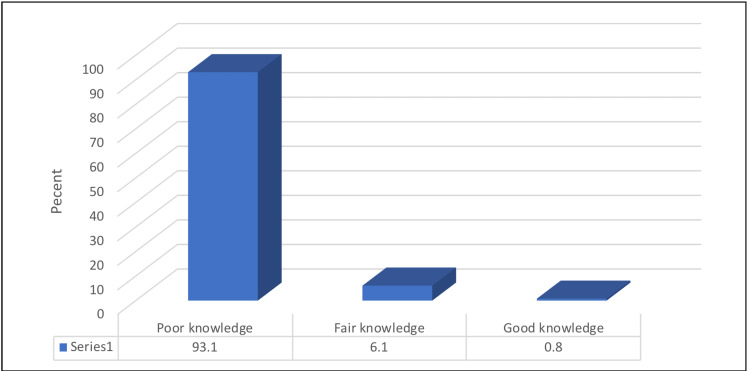
Percentage distribution of parents according to their level of knowledge about testicular torsion (n=394)

The most common sources of information about testicular torsion among participants were the internet (38.6%) and a friend (33%) as illustrated in Figure 3. The mean knowledge score was 4.36 ± 2.62 and based on the knowledge scores; 91.1%, 6.1%, and 0.8% of parents had poor, fair, and good knowledge about testicular torsion respectively (Figure 4). The good knowledge about TT was significantly higher among children's fathers compared to mothers (66.7% vs. 33.3%) (p≤0.05). On the other hand, a non-significant relationship was found between parents' knowledge level and other demographics or the number of daughters and sons (p≥0.05) (Table 3).

**Figure 5:**
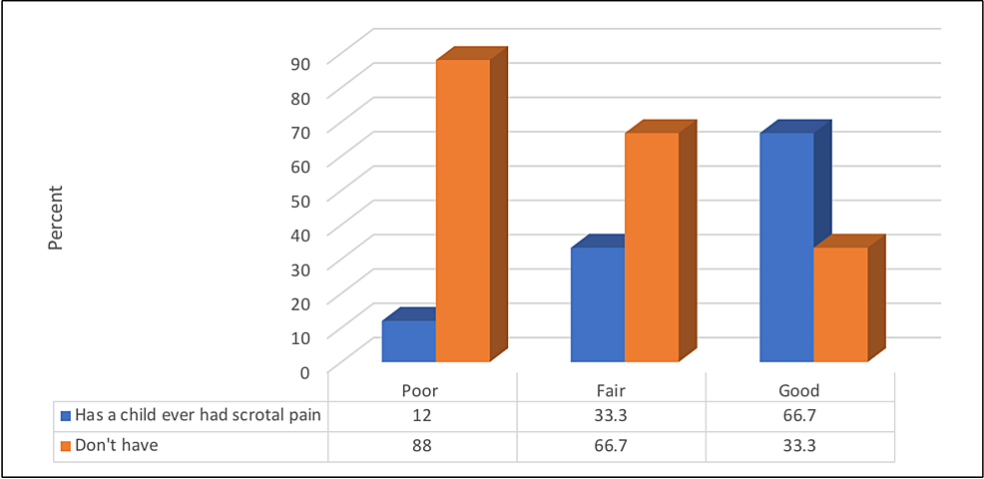
Relationship between parents' knowledge level about testicular torsion and having a child who had pain in the scrotum previously (n=394) N.B.: (χ2 = 9.84, p-value = 0.007)

**Table 3:** Relationship between parents' knowledge level about testicular torsion and their demographics, and the number of daughters and sons (n=394)

Variable		Knowledge level			χ2	p-value
		Poor No. (%)	Fair No. (%)	Good No. (%)		
Age	<30	87 (23.7)	5 (20.8)	2 (66.7)	5.16	0.27
	30-49	228 (62.1)	16 (66.7)	0 (0.0)		
	≥50	52 (14.2)	3 (12.5)	1 (33.3)		
Nationality	Saudi	334 (91)	22 (91.7)	3 (100)	0.3	0.858
	None-Saudi	33 (9)	2 (8.3)	0 (0.0)		
Relation to the child	Father	100 (27.2)	13 (54.2)	2 (66.7)	9.95	0.007
	Moher	267 (72.8)	11 (45.8)	1 (33.3)		
Education level	Illiterate	2 (0.5)	0 (0.0)	0 (0.0)	1.42	0.994
	Primary	2 (0.5)	0 (0.0)	0 (0.0)		
	Middle	16 (4.4)	1 (4.2)	0 (0.0)		
	Secondary	81 (22.1)	5 (20.8)	0 (0.0)		
	University	266 (72.5)	18 (75)	3 (100)		
Employment	No	151 (41.1)	6 (25)	0 (0.0)	4.45	0.108
	Yes	216 (58.9)	18 (75)	3 (100)		
The number of daughters	1-2	204 (55.6)	11 (45.8)	2 (66.7)	3.68	0.719
	3-4	89 (24.3)	9 (37.5)	0 (0.0)		
	≥5	16 (4.4)	1 (4.2)	0 (0.0)		
Number of sons	1-2	224 (61)	14 (58.3)	1 (33.3)	9.48	0.148
	3-4	87 (23.7)	10 (41.7)	2 (66.7)		
	≥5	13 (3.5)	0 (0.0)	0 (0.0)		

**Figure 6:**
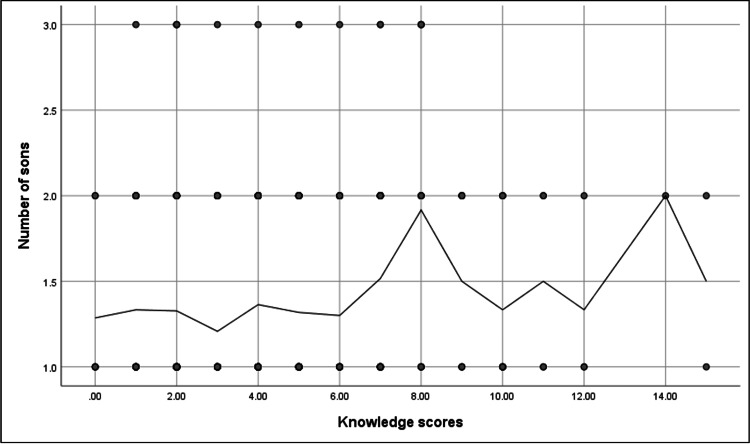
Spearman's correlation analysis between knowledge scores and the number of sons N.B.: (r = 0.13, p-value = 0.01)

It was demonstrated that good knowledge about TT was significantly higher among parents who reported having a child who had experienced scrotal pain before (p≤0.05) (Figure 5). In addition, a significant positive correlation was found between knowledge scores and the number of sons (r = 0.13, p-value = 0.01) as shown in Figure 6. 

## Discussion

Since there is limited evidence in the literature about parental awareness of TT in children, we found it crucial to survey parents to ascertain their awareness and attitude in such critical conditions. The level of awareness and understanding of testicular torsion among parents in the western region of Saudi Arabia were evaluated for the first time in this study.

Most of the time, parents or guardians are in charge of their children's health and well-being [1]; they owe them the responsibility to decide whether or not an urgent presentation to the emergency department is necessary. Studies showed that pediatric patients are more likely to have delayed presentation and subsequent complications if their parents have poor knowledge about their disease [10]. The study by Barada et al. has shed more light on the correlation between orchidectomy rates and patient age; it showed a significant difference when compared between pediatric patients under the age of 18 (44%) and adult patients beyond the age of 18 (8%) [13]. In addition, the Nevo et al. findings support those of Barada et al. which demonstrated that orchidectomy is significantly influenced by younger age [14]. According to Yap et al. study, there is a statistically significant correlation between parents' knowledge of testicular torsion and their likelihood of presenting immediately to ED in the setting of severe pain. Parents who were aware of testicular torsion were four times more likely to do so than those who were not, and parents who correctly identified the critical time frame were three times more likely to do so than those who did not [10]. Two-thirds of the testicular torsion patients in the Ubee et al. study presented six hours after the onset of symptoms [9]. In children with torsion, any factor influencing a delayed hospital presentation is crucial as it might be a key contributing factor in boys ending up having an orchidectomy. Our findings showed that only 17.8% of our participants knew that the appropriate time for surgical intervention is within four to six hours. Reduced delay at presentation might be achieved by raising community awareness of testicular torsion.

According to parents' responses to their children's testicular pain, our results determined that most of the parents correctly reported that if the child complains of pain in the scrotum during working hours (68.8%) or over the weekend (76.6%), they would drive him to the hospital immediately. Friedman et al. similarly observed from their data which targeted the parents in ear, nose, throat (ENT), and urology clinics, the parents in both clinics gave statistically similar answers (72.5% and 75%) regarding their responses during business hours for taking their children immediately to the emergency department or call their physician within one hour. Similar trends were reported in their response during the weekends (80.4% and 81.9%) [12]. These results are in line with those obtained by Alyami et al. who compare the awareness of pediatric urology clinics and general pediatric clinics among parents of children who have attended both clinics. The family's response was the same in all situations, with their decision to head straight to the emergency room being the most favorable [8]. In contrast, what particularly stands out is these results are not in line with those of Ubee et al. study which found only (22%) of his cohorts brought their children to the ER immediately. The remaining parents sought counsel from other sources, and some even waited all night for the medical facilities to open [9]. This attitude was comparable to Anzaoui et al.'s study, which showed that 72% of patients would arrive at hospitals late, six hours after the onset of pain [15]. All of these responses show that those parents are unaware of the severity of the illness and the potential consequences that could arise if immediate intervention is not provided, where studies have found that if a patient seeks medical attention within the first six hours of the onset of symptoms, their chance of having their testicles saved is over 100%; but it soon falls to less than 50% if more than 12 to 24 hours pass [6].

Our results are consistent with those obtained in the Alyami et al. study; it found that as the number of boys in the family increases, the parents' awareness of TT also increases [8]. Parents with only girls in the family will not directly be impacted, but if they were aware of it, they might share the knowledge with other parents who have boys in their family since several studies have demonstrated that parents who were aware of testicular torsion mostly learned about it via friends, relatives, and the internet, which stands as the most common source of knowledge [8-16]. 

The parent’s response about the indicator of a serious condition in our study was if the pain didn’t relieve in a short time (48%), while the parents in Alyami et al. study (46.95) responded that severe pain and the appearance of the testicle were indicators of a serious condition [8]. Our results demonstrate that the good knowledge about TT was significantly higher among children's fathers compared to mothers (66.7% vs. 33.3%); The father's prior experience with circumstances comparable to these or learning about the experiences of friends of the same sex might also provide an explanation of this significant difference in awareness between mothers and fathers. Moreover, according to our survey, a relatively high number of parents (93.1%) had poor knowledge about TT, which is also similar to Alyami et al. results [8]. Additionally, 55.1% of parents' responses in our study were not aware of the complications of TT. As part of the care we provide for our patients, we think that raising awareness of TT is essential to avoid its consequences. Educating the general Saudi population, particularly parents and young males, is warranted through targeted community-based campaigns and social media platforms. Focusing on raising awareness of testicular torsion among preadolescent and adolescent boys as well as their parents should result in a drop in pre-hospital delays, directly corresponding to a decrease in rates of testicular loss.

## Conclusions

Testicular torsion (TT) represents significant morbidity among male patients. Early identification is crucial to avoid the need for orchiectomy and all its prominent physical and psychological consequences. To improve children's well-being, we need to raise parents' awareness of TT and the potential future implications of this critical condition as it is not widely known in the surveyed community. By doing this, we can hopefully reduce prehospital delays and increase the likelihood that testicles can be saved. Further studies evaluating the knowledge regarding testicular torsion among preadolescent and adolescent boys are warranted.
